# Association of income relative deprivation and sleep duration in China

**DOI:** 10.3389/fpsyg.2022.1008259

**Published:** 2022-11-18

**Authors:** Zijian Peng, Lin Wu

**Affiliations:** School of Sociology, Wuhan University, Wuhan, China

**Keywords:** income relative deprivation, sleep duration, social trust, social mentality, instrumental variable method

## Abstract

In recent years, the rapid development of China’s economy has brought about a serious polarization between rich and poor, which makes people have to bear the impact of social changes on their physical and mental health while enjoying the benefits of social development. It is difficult to maintain normal sleep duration (7–9 h), which has gradually become a social phenomenon. Based on the China Family Panel Studies (CFPS2018), this study explored the relationship between relative income deprivation and sleep duration at the micro-level. This paper empirically tests that the probability of normal sleep duration (7–9 h) decreases by 22.8% for each unit of income relative deprivation. This conclusion is significant at 0.05 level. On this basis, the instrumental variable method is used to overcome the endogenous problem, and a more accurate conclusion is obtained. After the robustness test and heterogeneity analysis of the model, a mediation model is constructed through Mplus: relative income deprivation – social trust – sleep duration. Social trust is considered as a mediation variable. This study believes that in Chinese society, the relative deprivation of individual income will affect their sleep duration by changing their social trust. Therefore, increasing the income of low-income groups, narrowing the gap between rich and poor, alleviating social conflicts, and promoting interpersonal trust are important means to ensure that social members can have normal sleep duration.

## Introduction

Scientific research shows that an adult’s normal sleep duration should be between 7 and 9 h, less than 7 h is insufficient sleep duration, and more than 9 h is too long sleep duration (Hirshkowitz et al., 2015; Ren et al., 2019). Sleep duration of 7–9 h is considered to play an important role in maintaining normal physiological functions and improving quality of life, and it is considered to be one of the important factors for individual physical and mental health and life happiness (Yan et al., 2020). According to the statistics of China Sleep Research Society (CSRS), with the increased pressure and pace of life, the average sleep duration of Chinese people has dropped from 8.8 to 6.5 h from 2013 to 2018. During the working day, only 67.24% of people suffer from sleep disorders (Yueqiu et al., 2022).

Sleep duration less than 7 h or more than 9 h is considered abnormal sleep duration, which will lead to unhealthy health (Steptoe, 2006; Stranges et al., 2008): increased probability of cardiovascular and cerebrovascular diseases, diabetes, heart disease (Christa et al., 2007), and obesity (Vgontzas et al., 2008; Peltzer and Pengpid, 2017; Fan et al., 2020); cognitive decline and dementia risk is greatly increased (Cappuccio et al., 2010; Chien et al., 2010). The researchers attribute the poor health caused by the short or long sleep duration mentioned above to the individual’s physical qualities such as glucose tolerance, insulin sensitivity, hunger, and endocrine function (Van Cauter and Knutson, 2008); living habits such as sleep patterns, drug intake, eating habits (Genderson et al., 2014); disease history such as coronary heart disease, myocardial infarction, and various physical discomfort (Peltzer and Pengpid, 2017; Ren et al., 2019).

From the above analysis, we can see that the previous research on sleep duration was mostly focused on the biomedical field, but more and more research began to focus on the role of social factors in sleep duration, especially the impact of income. A large number of empirical studies have shown that absolute income is an important factor affecting health (Guoqiang et al., 2017). As an important measure of health, whether sleep duration is normal is also affected by absolute income (Ancoliisrael et al., 1991). Heslop et al. (2002) proposed that workers with lower income usually have longer working hours to improve their living conditions, which will greatly reduce their sleep time. Stamatakis et al. (2007) conducted a longitudinal study on the sleep duration of the population in California, the United States, and found that socio-economic status is a strong determinant of short sleep duration, especially when the family income is low, individuals have a higher probability of shorter sleep duration. Some studies have pointed out that the social groups with higher income have the ability to pay for various late night entertainment activities, such as the Internet, television, and party activities, so the individuals with higher income sleep longer than the individuals with lower income (Bliwise, 1996). It can be seen that although the relevant research did not reach an agreement on the relationship between income and sleep duration, it proved that income did have an impact on sleep duration.

Most of the previous studies focused on the direct role of absolute income in people’s sleep duration, and lack of research on the role of relative income in sleep duration. As another form of income, relative income has a significant impact on social members (Deaton, 2001). Wilkinson (1996) pointed out that relative income has a greater impact on people’s health and living conditions than absolute income, which well explains the relationship between income inequality at the individual level and their health and life. In daily life, people not only pay attention to their own income but also pay attention to the income of others and compare it with their income. The theory of relative deprivation of income proposed by Townsend (1979) points out that people tend to make upward social comparisons, that is, to compare themselves with people with better income status. In this process, individuals will face the income gap with others directly and thus produce income relative deprivation. Therefore, relative deprivation of income is considered as a measure of individual income inequality. When individuals face the same income inequality, their subjective feelings of deprivation are not the same. Especially in China, the economic boom brought about by the reform and opening up has caused a serious imbalance in social development, which makes people enjoy social progress, but also face the costs and pain brought about by social transformation. Health is facing unprecedented challenges (Jiawen, 2016). Insufficient sleep has become a widespread social phenomenon. According to the income inequality hypothesis, as the gap between the rich and the poor in society gradually expands, it not only increases the number of relatively poor people but also makes the whole social group feel strongly deprived (Adjaye-Gbewonyo and Kawachi, 2012). Therefore, under the social background of severe social differentiation and the gap between rich and poor brought about by China’s social and economic development, the Chinese people’s sense of income relative deprivation is widespread. This shows that under the background of widespread income deprivation and insufficient sleep in China, it is of great theoretical and practical significance to explore the impact of income relative deprivation on sleep duration and its mechanism.

### Income relative deprivation and sleep duration

Since the late 1990s, more and more studies have begun to pay attention to the impact of individual income inequality on people at the micro-level. According to the theory of relative deprivation, people always tend to make upward social comparisons with individuals who are higher than their own social economy, rather than lower (Runciman, 1966). As an important component of socio-economic status, income is a key indicator to measure the relative deprivation of micro-individuals. Income relative deprivation describes the impact of income inequality on different individuals. Through sorting out relevant studies, the mechanism of relative income deprivation affecting sleep duration can be summarized in the following two levels.

First of all, income relative deprivation affects the sleep duration of individuals through material channels. In general, the lower the income level of individuals, the higher their income relative deprivation (Mangyo and Park, 2011; Gunasekara et al., 2013). That is, the higher the degree of income relative deprivation, the lower their income level and the poorer they are compared with others. The high degree of income relative deprivation means the relative poverty on the material level. Crimmins et al. (2009) found that people living in poverty are often accompanied by sleep problems, such as insufficient sleep. The reasons why income relative deprivation affects individual sleep duration through material channels can be attributed to the following two reasons. First, individuals with high degree of income relative deprivation have low income, poor living environment, and short sleep duration. Adler and Ostrove (1999) pointed out that compared with low-income groups, high-income groups have a better living environment. Their living area is large, the environment is good, and temperature and noise are well controlled. Their sleeping space is superior. Therefore, the high-income group has a shorter sleep duration than the low-income group, and is more likely to have a longer sleep duration (Lauderdale et al., 2006). Second, individuals with a high degree of income relative deprivation have a low income, and it is difficult to obtain material resources to maintain health, which will affect their sleep duration. Poor health is an important factor that aggravates sleep disorders and is not conducive to maintaining normal sleep duration (Stamatakis et al., 2007). Many studies have pointed out that the income relative deprivation has a negative impact on people’s health (Basta et al., 2007; Akay et al., 2018). This is because the income of low-income groups is difficult to support them to obtain basic medical services and purchase other health insurance, which makes them vulnerable to poor health (Mangyo and Park, 2011). Lutfey and Freese (2005) conducted a one-year ethnographic survey on outpatients with diabetes and found that most of the specific mechanisms affecting patients and obtaining high-quality medical resources and services are more favorable to patients with high socioeconomic status. Therefore, the higher the degree of income relative deprivation, the shorter their sleep duration.

Secondly, income relative deprivation affects the sleep duration of individuals through psychological channels. The theory of relative deprivation points out that because people always have an upward social preference, the low-income group has a stronger sense of income relative deprivation than the higher income group, which will cause the low-income group to have a persistent sense of inefficiency (Eibner and Evans, 2005). This negative emotion makes them constantly produce stress and anxiety, which is the key factor to shorten the sleep duration (Yan et al., 2018). There are two reasons why income relative deprivation affects individual sleep duration through psychological channels. First, low-income groups are often in a lower socio-economic status in society, which makes them have a higher level of stress and anxiety than high-income groups. The continuous negative psychological state will make low-income groups with a higher sense of deprivation more likely to fall into depression, which is not conducive to sleep (Thomsen et al., 2003). Stewart et al. (2011) pointed out that the higher a person’s anxiety test score, the greater the likelihood of insufficient sleep. It can be seen that higher stress levels cannot be ignored in disturbing sleep duration (Kerstedt, 2006). Second, compared with high-income groups, low-income groups have less social resources. They always feel lack of resources when solving problems and thus generate huge psychological pressure, which will also directly lead to sleep deprivation in low-income groups with a higher sense of deprivation (Barber et al., 2013). Hardie and Lucas (2010) pointed out that the income relative deprivation will lead to the decline of marriage quality, increase the frequency of family conflict and domestic violence, and make people feel less social support when dealing with daily problems, increasing the pressure level of family members in family relations. Therefore, the higher the degree of income relative deprivation, the shorter their sleep duration.

### Social trust as an intermediary variable

Based on the above analysis, this paper constructs an explanation framework between income relative deprivation and sleep duration. However, we cannot ignore that sleep duration is determined by complex social processes. In this process, people allocate and negotiate their sleep duration of the day according to their social status, social roles, and resources (Burgard, 2011). Therefore, this paper tries to find the mediating mechanism of income relative deprivation affecting sleep duration. Bai and Luo (2014) pointed out that the trust crisis caused by the polarization between the rich and the poor in China today is an important problem faced by the society. According to the data of the World Values Survey, the level of social trust of Chinese people is declining year by year. The social and economic comparison at the individual level caused by the income gap has aggravated their sense of deprivation, led to bad social relations and interpersonal distrust, had a negative impact on the physical and mental health of individuals, and made it difficult for people to have normal sleep duration. First, the income relative deprivation has a negative impact on social trust. According to the principle of homogeneity preference, people with the same income status communicate more frequently. The worsening income inequality has strengthened the difference in social status, led to estrangement and alienation between people, and significantly reduced social trust (Yiwei, 2018). Many studies have emphasized that there is a highly negative correlation between income inequality caused by the polarization of the rich and the poor and people’s social trust (Uslaner, 2005; Freitag and Buhlmann, 2009). Brehm and Rahn (1997) estimated a structural model using data from 1975 to 1994, including citizen participation, social trust, and government confidence. They found that Gini coefficient would significantly reduce people’s social trust. Eliana and Alesina (2000) used the census data to calculate the Gini coefficient to measure the income gap, which also proved the negative impact of income heterogeneity on social trust. Guangjun and Chuanchuan (2016) studied the income gap and social trust in China, and found that under the realistic background of the sharp decline of social trust level and the rapid deterioration of income distribution pattern in contemporary China, the social differentiation caused by income gap is an important factor leading to the reduction of trust among social members. Secondly, low social trust makes it difficult for individuals to maintain normal sleep duration. The improvement of social trust can increase the social support and social participation of individuals, thus effectively weakening the various chronic pressures they bear (Sato et al., 2018; Saura et al., 2021). A high degree of social trust makes people not need to keep tense all the time, enhances their self-esteem and confidence when dealing with things, and reduces the possibility of negative emotions through emotional support (Mikucka et al., 2017). A positive emotional state helps people maintain a normal sleep duration. Numerous studies have proved this: Japanese scholars Sugawara et al. (2020) pointed out in a study on social trust and sleep that low socio-economic status and poor living standards will reduce people’s social trust, increase their stress level, and damage their health, and they are usually difficult to maintain normal sleep duration. Chinese scholar Jiawen (2016) proposed in his research on income inequality and personal well-being in Chinese society that social trust plays a mediating role between income inequality and personal well-being. As a subjective psychological emotion, personal happiness will affect people’s sleep. Kawachi et al. (1997) pointed out that the lower the level of social trust, the higher the possibility of increased mortality, which has a negative impact on people’s health and is difficult to maintain a normal sleep duration. Therefore, this paper argues that social trust is the intermediary mechanism that explains the relationship between income relative deprivation and sleep duration.

After sorting out the existing research, it is found that the existing literature lacks the research and mechanism exploration of the direct role of income relative deprivation in sleep duration. And many studies are based on the analysis of the phenomenon of developed countries. However, the research based on the background of developed countries is not suitable for the actual situation of China. As a large developing country with rapid social changes, the rapid development of China’s economy has gradually worsened the income pattern and seriously challenged the national spiritual life. Based on the above considerations, this paper uses China Family Panel Studies (CFPS2018) data to study the impact of income relative deprivation on the sleep duration of social members and analyze the impact path under the Chinese background. The innovations of this paper are as follows: First, in terms of research perspective, this paper uses the individual level income inequality index Kakwani (1984) to measure the income relative deprivation, which is used to measure the impact of income inequality on different individuals and overcome the problem that other indexes are sensitive to income scale. It is included in the overall analysis framework between income difference and sleep duration of Chinese residents, revealing the relationship between income inequality and sleep duration from a micro-individual perspective. Second, in terms of research methods, on the basis of overcoming the endogenous problem with instrumental variable method, this paper analyzes the heterogeneity from three dimensions of region, marital status, and housing quantity. The mechanism of the effect of income relative deprivation on sleep duration was explored by intermediary analysis. It provides a more comprehensive perspective to understand the relationship between income relative deprivation and sleep duration in the Chinese context.

## Research questions and assumptions

In order to overcome the above shortcomings of previous empirical studies, this study explored and analyzed the changes and mechanisms of sleep duration of Chinese people with Kakwani (1984) income relative deprivation index under the background of China’s reality, using China Family Panel Studies (CFPS2018) data, and from the perspective of relative deprivation at the micro-level. After combing the realistic background and existing literature in the introduction, this paper proposes the following two assumptions:

Hypothesis 1: There is a negative correlation between income relative deprivation and whether normal sleep duration can be maintained. The higher the degree of income relative deprivation, the more difficult it is to maintain normal sleep duration; on the contrary, the lower the income relative deprivation, the easier it is to maintain normal sleep duration.

Hypothesis 2: Social trust is a mediator between income relative deprivation and whether normal sleep duration can be maintained. The income relative deprivation affects their sleep duration by affecting their social trust.

## Data and methods

### Data and samples

This study uses China Family Panel Studies (CFPS2018), which is a large-scale national social survey project carried out by Institute of Social Science Survey (ISSS). CFPS aims to reflect the changes of China’s society, economy, population, education, and health by tracking and collecting data at three levels: individual, family, and community. It is a national, large-scale, and multidisciplinary social tracking survey project. The baseline survey was carried out in 2010, and the tracking survey was carried out every two years thereafter. Taking into account the regional differences in Chinese society, in order to save survey costs and improve the representativeness and scientificity of sample sampling, CFPS adopts a multi-stage, implicit stratified sampling method (PPS) proportional to the size of the population. Because of the scientific and authoritative nature of CFPS in China, the use of CFPS data to study Chinese society has been recognized and adopted by an increasing number of Chinese scholars (Yu et al., 2014).

CFPS2018 has five types of questionnaires: family members’ questionnaire, family economic questionnaire, individual self-administered questionnaire, children’s parents’ proxy questionnaire, and individual proxy questionnaire, covering all family members in families and sample households in 25 provinces, cities, and autonomous regions in China. The total sample size is 12,421 families and 32,669 individuals. Due to the calculation of the kakwani income relative deprivation index, the object of this study is adults with income. In order to establish an appropriate database, the following steps were carried out: First, use Stata software to select the appropriate variables in the CFPS2018 family database and adult database, and combine them. Second, the missing values, singular values, and interrupted samples are eliminated, and the samples with income of 0 are excluded. Only adult samples with income above 0 are retained. Third, sorting out the selected variables according to the research needs. After the above steps, 8650 valid samples were obtained.

### Variable setting

#### Dependent variable

The dependent variable of this study is “sleep duration”. In adults with work and income, the sleep duration usually depends on the schedule of the working day, so the sleep duration of the working day can better reflect the individual’s regular living conditions (Dinges et al., 2006). Therefore, this paper chooses the question of sleep duration in CFPS2018, “Generally speaking, you sleep several hours every day on weekdays,” to calculate the dependent variable. According to previous studies, 7–9 h is normal sleep duration, less than 7 h is insufficient sleep, and more than 9 h is too long sleep (Hirshkowitz et al., 2015; Ren et al., 2019). We learned from Stamatakis et al. (2007) treated sleep duration as a categorical variable: 7–9 h of sleep duration was normal sleep duration, and less than 7 h and more than 9 h of sleep duration were abnormal sleep duration.

#### Independent variable

The independent variable of this study is “income relative deprivation.” Since the theory of relative deprivation was put forward, the measurement of relative deprivation has become the focus of academic discussion, and many representative measurement indicators have emerged. For example, Yitzhaki index, Kakwani index, Podder index, Esposito index, and relative deprivation index are considered as the range of income values. Kakwani index satisfies the good properties of dimensionless, normal, and transfer invariance. Therefore, this paper uses the kakwani income relative deprivation index proposed by Kakwani (1984) in to measure the different negative effects of income inequality on different individuals.

#### Instrumental variables and mediating variable

In order to overcome the possible endogenous problems of income relative deprivation and sleep duration, this study calculated the “average income relative deprivation index of the same village/household” as a tool variable according to the relative income deprivation index. As the economic income development of each region has obvious spatial and geographical effects, the income level of people in the same region usually depends on the economic development level of the region. Therefore, there is a strong correlation between the individual income relative deprivation index and the average income relative deprivation index of the same village/resident, but the correlation between the average income relative deprivation index of the same village and the individual is weak. In addition, in order to explore the specific mechanism of income relative deprivation affecting individual sleep duration, this study set up the intermediary variable of social trust. Drawing on previous research using CFPS data to measure social trust, construct a virtual variable of social trust based on the question “Generally speaking, do you think most people can be trusted, or should you be as careful as possible to get along with others”: when respondents answer “most people can be trusted,” it is 1; when the respondent answered “The more careful, the better,” it was 0 (Guangjun and Chuanchuan, 2016).

#### Control variable

In order to make the conclusion of the relationship between individual income relative deprivation and sleep duration more reliable, and reduce the problem of missing variables as much as possible, we set up several control variables from the individual, family, and regional levels, and reduce the problem of missing variables as much as possible, we set up several control variables from the individual, family, and regional levels. Individual level characteristics: gender, registered residence, education, health, whether or not a member. Family level characteristics: housing property rights, family population, whether there are two or more houses, the total real estate value of the family, the net income of the family. Regional characteristics: region, regional average household income. The specific meanings of these control variables are shown in Table 1.

**TABLE 1:** Key variable description statistics.

Vartype	Varname	Mean/Frequency	SD/Percentages	Min	Max	Obs
Dependent variable	sleep duration	0.729	0.444	0	1	8650
Independent variable	kakwani	0.047	0.056	0	0.273	8650
Control variable	gender	3843	0.444	0	1	8650
	hukou	3171	0.367	0	1	8650
	edu	0.232	0.422	0	1	8650
	health	3.240	1.088	1	5	8650
	cpc	8485	0.981	0	1	8650
	hproperty	1401	0.162	0	1	8650
	sfamily	4.093	1.989	1	17	8650
	nhouse	6604	0.763	0	1	8650
	ln_fhousem	12.628	1.431	9	16	8650
	ln_fincome	11.332	0.701	10	13	8650
	re_ln_fincome	11.333	0.130	11.149	11.460	8650
Other variable	mkakwani	0.047	0.029	0	0.273	8650
	trust	0.576	0.494	0	1	8650

The frequency and percentage of categorical variables in this table are calculated based on category 0.

### Empirical strategy

Based on the core research content, the explained variable of this paper is sleep duration. It is binary dummy variables. Therefore, this paper selects Probit model which can deal with binary variables for regression analysis. The model settings are as follows:


(1) $$\text{Probit}\,\left({\text{Sleep}}_{\text{i}}\right)=f\,\left(\beta_{1}\,{\text{Inequality}}_{\text{i}}+\beta_{2}\,{\text{X}}_{\text{i}}+\varepsilon_{\text{i}}\right)$$

In Equation (1), Sleep_i_ is the binary variable of sleep duration, subscript i represents the ith individual, Inequality_i_ represents the individual income relative deprivation measured by Kakwani relative deprivation index, and ε_i_ represents the random disturbance term. In order to reduce the missing variables in the model, X_i_ is introduced to represent other control variables in this paper.

Because the estimation in Equation (1) may have endogenous problems, some potential factors may affect the individual income relative deprivation and the sleep duration at the same time due to measurement errors, missing variables, and reverse causality. However, we cannot effectively control these factors in Probit model, so the estimation result of individual income relative deprivation on sleep duration in the model may be biased. In order to deal with the potential endogenous problem, we used the instrumental variable method to re-estimate (1) and then carry out IV Probit two-stage regression on this basis. In the first stage, we used the explanatory variable inequality to perform OLS regression on other variables to obtain the fitting values of residual $\hat{\varepsilon_{\text{i}}}$ and latent variable Inequality*:


(2) $${\text{Inequality}}_{\text{i}}^{*}=\mu_{0}+\mu_{1}\,\vec{\text{Z}}+\mu_{2}\,{\text{X}}_{\text{i}}+\varepsilon_{\text{i}}⟹\hat{{\text{Inequality}}_{\text{i}}^{*}}$$

In Equation 2, $\vec{\text{Z}}$ represents the tool variable, Inequalityi*^ represents the fitting value of Inequality*. X_*i*_ is the same control variable as in Probit model. In the second stage, the explained variable sleep performs Probit regression on the fitting value, residual and exogenous explanatory variables of the latent variables, and the consistent estimated value is obtained. The expression is:


(3) $${\text{Sleep}}_{\text{i}}^{*}=\alpha_{\text{i}}+\beta_{\text{i}}^{*}\,\hat{{\text{Inequality}}_{\text{i}}^{*}}+\gamma \,{\text{X}}_{\text{i}}+\lambda_{\text{i}}+\xi_{\text{i}}$$

In Modern Econometrics, $\vec{\text{Z}}$ must meet the following two conditions: first, exogenous cov($\vec{\text{Z}},\xi_{\text{i}}$) = 0. Second, it is significantly correlated with endogenous variable Inequality. The instrumental variables selected in this paper and its effectiveness test are described in detail below.

## Results

### Descriptive statistics

Table 1 presents the descriptive statistics of the key variables used in this paper. It can be seen that in the samples we used, nearly 30% of Chinese adults aged 16–60 do not have normal sleep duration. The average of individual income relative deprivation index is 0.047. From the individual level of the interviewed group, it can be found that men account for 59%, agricultural registered residence accounts for 70%, and the proportion of people who have received university and above is 23%, and the proportion of Party members is 0.2%. This is also more consistent with China’s basic national conditions, such as the large number of male population, large rural population and low popularity of higher education. From the family level of the respondents, it can be found that the average number of family members is 4, which is basically consistent with the effect of China’s family planning policy. 84% of the households have their own housing property rights, and only 24% of the households have second or more houses. The standard deviations of the total house property value (logarithm) and the net family income (logarithm) are 1.431 and 0.701, respectively. There is great heterogeneity of property among families. These data are in line with the reality of China’s large gap between the rich and the poor and unequal distribution of income and wealth.

### Correlation results

In this paper, Probit model is used to analyze the impact of individual income relative deprivation on sleep duration. We used the method of gradually increasing control variables to observe the fitting degree of the model. In Table 2, model 1 shows the marginal effect of Probit regression without adding control variables, and model 2 shows the marginal effect of Probit regression with adding control variables. According to the regression results of model 1, the effect of income relative deprivation on sleep duration was significant at the level of 1%. Model 1 points out that without adding control variables, income relative deprivation is not conducive to maintaining a normal sleep duration (7–9 h). For each unit of increase in people’s income relative deprivation, the probability of a normal sleep duration (7–9 h) decreases by 27.8%. It may be that when people make an upward social comparison of income, the sense of relative deprivation makes them have more emotional stress and dissatisfaction with the current situation of life. This negative emotion is not conducive to have normal sleep duration (7–9 h). Considering that other factors may also affect people’s sleep duration, we added control variables in model 2. With the increase of variables, the impact of individual income relative deprivation on normal sleep duration (7–9 h) decreased compared with model 1, but it was still significant at the level of 5%. After adding the control variable, the probability of having normal sleep duration (7–9 h) decreased by 22.8% for each unit of increase in people’s income relative deprivation. The control variables basically display the direction symbols consistent with the expectation. At the individual level, men, rural residents, individuals with partners, individuals with low education and individuals with poor health are accompanied by short or long sleep duration. At the family level, there is a significant negative correlation between family net income and having normal sleep duration, and the total real estate value of the family has a positive impact on having normal sleep duration (7–9 h). Compared with model 1, r2 increases from 0.001 to 0.014 after adding control variables, which shows that the goodness of fit of model 2 is significantly improved, fully reflecting the rationality of model selection. This conclusion supports hypothesis 1 of the text.

**TABLE 2:** Correlation between income relative deprivation and rationality of sleep duration.

Explanatory variable	Model 1	Model 2
	Marginal effect	Marginal effect
Kakwani	–0.278***	−0.228**
	(–3.35)	(−2.46)
Gender		−0.031***
		(−3.04)
Hukou		0.029**
		(2.45)
Marriage		−0.024*
		(−1.91)
Edu		0.089***
		(6.79)
Health		0.027***
		(6.28)
Cpc		0.027
		(0.76)
Hproperty		–0.003
		(−0.25)
Nhouse		–0.007
		(−0.54)
Sfamily		0.003
		(0.94)
ln_fincome		−0.015*
		(−1.67)
ln_fhousem		0.011***
		(2.60)
re_ln_fincome		–0.307
		(−0.77)
Regional fixed effect	No	Yes
_cons	0.651***	10.953
	(34.52)	(0.80)
N	8650	8650
r2	0.001	0.014

*p < 0.1; **p < 0.05; ***p < 0.001.

### Endogenous problem

In order to deal with the possible endogenous problem of income relative deprivation, we re-estimated the regression results of Probit model by instrumental variable method IV Probit. After controlling other variables, Table 3 presents the results of re-estimation of the model, in which the second column is the estimation results of the first stage and the third column is the estimation results of the second stage. After estimating the model, we tested whether the instrumental variable “average individual income relative deprivation index of the same village/residence” has the problem of weak instrumental variables. The results show that in the first stage, *f* = 337.05, far greater than 10. The relationship between instrumental variables and explanatory variables is significant at the level of 1%. Therefore, it can be determined that there is a significant strong correlation between instrumental variables and explanatory variables. At the same time, according to the previous theoretical assumptions, it is difficult to establish a correlation between the average individual income relative deprivation index of the same village/residence and the sleep duration. And in our data analysis results, it also shows that there is no correlation between them. In this sense, we believe that “relative deprivation of average individual income in the same village/residence” is a reasonable instrumental variable. This is also confirmed by the estimation results in Table 3. The estimation results of the first stage show that “relative deprivation of average individual income in the same village / residence” has a significant positive impact on the explanatory variable “individual income relative deprivation”. In the first stage, the goodness of fit of the model is ideal. The results of the second stage show that after we use the instrumental variable method to re-estimate the model, the higher the income relative deprivation, the lower the probability of having normal sleep duration. The relationship is significant at the 5% level, and the correlation estimation coefficient of the model does not change much. At the same time, Wald and Ar are both significant at the 1% level, indicating that the instrumental variables selected in this paper are not weak instrumental variables. Compared with Probit model, the result of re-estimation by instrumental variable method is more accurate and reliable. For the control variables of the model, the estimation results are basically consistent with those of Probit model, which will not be repeated here.

**TABLE 3:** Instrumental variable estimation results.

Variable name	IV Probit	
	First-stage	Two-step
Mkakwani	0.824***	
	(43.80)	
Kakwani		–1.401**
		(–2.10)
_cons	0.246	12.225
	(0.54)	(0.89)
Control variable	Yes	Yes
Wald		4.39**
AR		4.39**
F	337.05	
r2	0.353	
N	8650	8650

**p < 0.05; ***p < 0.001.

### Robustness check

The above results show that there is a significant causal relationship between individual income relative deprivation and their sleep duration. With the increase of individual income relative deprivation, their probability of having a normal sleep duration will be reduced. In order to further test the robustness of the estimation results, we use three methods: changing independent variable, changing dependent variable, and changing models.

First, change independent variable. In Probit model, we measured the relative deprivation index of income through the annual wage income of individuals. Some studies have shown that income depends not only on individual labor market returns but also on family intergenerational assets transmission. Family assets is an important reason for individual income gap, it is particularly evident in China (Yi et al., 2017). In order to more accurately identify the impact of income relative deprivation on individual sleep duration, we change the unit of explanatory variable from individual to family, and measure the income relative deprivation index with family assets. In CFPS2018, the variables related to family assets include household durable goods consumption, total household agricultural machinery, household cash and deposits, household time deposits, and the total value of household financial products. After combining these five variables, the relative deprivation index of family assets is obtained. The instrumental variable method is used to re-estimate the model, and the results in the first column of Table 4 are obtained. The results show that when other variables are controlled, there is still a significant correlation between the relative deprivation of family assets and the sleep duration. The higher the relative deprivation of family assets, the lower the probability of having a normal sleep duration.

**TABLE 4:** Robustness check results.

Variable name	Model 1	Model 2	Model 3
	Replace independent variable	Replace dependent variable	Replace model
Fkakwani	–6.912***		
	(–3.69)		
Kakwani		–1.107*	–1.044**
		(–1.65)	(–2.13)
_cons	–8.336	29.199**	12.988
	(–0.63)	(2.17)	(0.55)
Control variable	Yes	Yes	Yes
N	8127	8117	8117

*p < 0.1; **p < 0.05; ***p < 0.001.

Second, change-dependent variable. Since the schedule of working days can better reflect the routine life state of individuals, we set up the sleep duration of working days as the explained variable in Probit model. However, some studies have suggested that because the state of individuals on working days is continuous, the sleep duration on rest days can also reflect their daily state (Akay et al., 2019). Therefore, we combine the sleep duration on rest days with the sleep duration on working days to obtain the individual’s total sleep duration as a new explained variable. The instrumental variable method is used to re-estimate the model, and the results in the second column of Table 4 are obtained. The results show that when other variables are controlled, individual income relative deprivation still has an impact on their sleep duration. The higher people’s income relative deprivation, the lower their probability of having normal sleep duration.

Third, change the model. Since the self-explanatory variable sleep duration is a binary dummy variable, the Probit model is selected as the main model in this paper. However, not only Probit model can deal with binary dummy variables but also Logit model can carry out regression analysis on such data. Therefore, we changed the model to Logit model for regression analysis, and obtained the results in the third column of Table 4. The results showed that when other variables were controlled, the individual income relative deprivation is still significantly negatively correlated with the sleep duration. The higher people’s income relative deprivation, the lower their probability of having normal sleep duration. This is consistent with the conclusion of Probit model.

### Heterogeneity analysis

In order to investigate the conditions under which individual income relative deprivation will affect sleep duration, we conducted a heterogeneity analysis on the sample. Table 5 presents the heterogeneity analysis results of the impact of individual income relative deprivation on sleep duration. First, after controlling for other variables, only when individuals are in eastern China, relative income deprivation is not conducive to individuals having normal sleep duration. Whereas in Western and central China, the effect of income relative deprivation on the sleep duration disappears. With economic development, China’s regional development gap is widening. The economic development level of the western and central regions is lower than that of the eastern region, and the per capita income of the central and western regions is significantly lower than that of the eastern region. The heterogeneity of per capita income in the eastern region is significantly higher than that in the central and western regions (Chaofang, 2012). Therefore, compared with the central and western regions, individuals in the eastern region will have a more obvious sense of income relative deprivation, which directly has a negative impact on the individuals having normal sleep duration. Second, after controlling for other variables, when respondents have partners, relative income deprivation is not conducive to individuals having normal sleep duration. The correlation between income relative deprivation and sleep duration disappeared when respondents had no partners. Studies have pointed out that when people get married or have a partner, they will be forced to have a stronger sense of responsibility and bear more pressure than when they are single (Xiaodong, 2020). Stress is not conducive to individuals falling asleep normally. Third, after controlling for other variables, when the respondent’s family owns two or more houses, relative income deprivation is not conducive to individuals having normal sleep duration. The correlation between relative income deprivation and sleep duration disappeared when respondents had only one house or no house. Some studies have pointed out that the increase of the number of houses will promote the risk investment of families. The increase of risk investment will increase the stress level of individuals and families, and then have a negative impact on having normal sleep duration (Kerdrain, 2011). It should be pointed out that in China, due to the huge differences in regional economic development, there are great differences in housing value, quantity, area, and quality. The housing unit price in underdeveloped rural areas is far lower than that in developed cities. Therefore, the large number of family houses does not mean that the total value of houses is also high. This explains why the negative impact of the number of houses in the heterogeneity analysis is different from the positive impact of the total value of real estate in the Probit model.

**TABLE 5:** Heterogeneity analysis results.

Variable name	Region			Marriage		Nhouse	
	West	Central	East	Have a partner	No partner	Two or more houses	No
Kakwani	–0.673	–0.787	–2.294**	–1.523**	–0.913	–2.525*	–1.090
	(–0.46)	(–0.67)	(–2.36)	(–1.99)	(–0.64)	(-1.86)	(–1.42)
_cons	0.306	1.135	0.703	3.776	51.206	–23.847	22.406
	(0.39)	(1.48)	(1.33)	(0.25)	(1.61)	(–0.82)	(1.43)
Control variable	Yes	Yes	Yes	Yes	Yes	Yes	Yes
N	2133	2405	4112	6764	1886	2046	6604

*p < 0.1; **p < 0.05; ***p < 0.001.

## Mechanism

**FIGURE 1:**
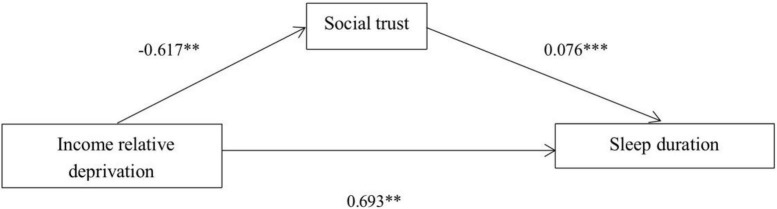
Mediation model of social trust. *p < 0.1; ^**^p < 0.05; ^***^p < 0.001.

Why does the lower income relative deprivation make people sleep more normally? In order to answer this question, this paper further analyzes the potential mediating mechanism that income relative deprivation may affect the change of sleep duration. Specifically, this paper constructs a mediation model through Mplus: income relative deprivation – social trust – sleep duration, and uses the bias correction percentile Bootstrap method to test the mediation effect. It can be seen from Figure 1 and Table 6 that social trust as an intermediary variable is effectively supported by data, and the total effect, direct effect and indirect effect are also statistically significant. The confidence interval of the total effect of income relative deprivation on sleep duration was (−1.243, −0.142), excluding 0, indicating that the total effect was significant. On the basis of controlling other variables, the confidence interval of the direct effect of income relative deprivation on sleep duration is (−1.136, −0.058), excluding 0, indicating that the direct effect is significant. The indirect confidence interval of income relative deprivation on sleep duration and hunger is (−0.008, −0.002), excluding 0, indicating that the indirect effect is significant. The path of intermediary effect can be understood as: the effect of income relative deprivation on social trust is −0.617, which is significant at the level of 0.05. The effect of social trust on sleep duration was −0.076, which was significant at 0.001 level. The effect of income relative deprivation on sleep duration is −0693, which is significant at the level of 0.05. To sum up, we believe that social trust is an important influence mechanism to explain the relationship between income relative deprivation and sleep duration. The income relative deprivation has a negative impact on the normal sleep duration by negatively affecting the social trust of individuals. This conclusion supports hypothesis 2 of the text.

**TABLE 6:** Analysis results of mediating effect.

Effect value	Effect quantity	Bootstrap 95% confidence interval
Total effect	–0.693**	(–1.243, –0.142)
Direct effect	–0.617**	(–1.136, –0.058)
Indirect effect	–0.076***	(–0.008, –0.002)

**p < 0.05; ***p < 0.001.

## Conclusion

This paper argues that in the context of the increasingly serious gap between the rich and the poor and the widespread lack of sleep in China, the individual income relative deprivation is the key factor affecting their inability to maintain normal sleep duration. Based on this, this paper uses the data of CFPS2018 to analyze the relationship between individual income relative deprivation and whether they can have normal sleep duration, and explores the mechanism. After a systematic review of previous relevant literature studies, we proposed two hypotheses for this study.

First, in order to answer the question of Hypothesis 1 “There is a negative correlation between income relative deprivation and whether normal sleep duration can be maintained. The higher the degree of income relative deprivation, the more difficult it is to maintain normal sleep duration; on the contrary, the lower the income relative deprivation, the easier it is to maintain normal sleep duration.” Based on the requirements of data types, we set the Probit model as the main model of this study. The results of Probit model show that there is a significant negative correlation between income relative deprivation and whether or not they have normal sleep duration. That is, the probability of normal sleep duration decreases by 22.8% for each unit of income relative deprivation. This conclusion is still valid after the endogenous problem is overcome and the model is re estimated using the instrumental variable method.

Second, in order to answer the question of Hypothesis 2 “Social trust is a mediator between income relative deprivation and whether normal sleep duration can be maintained. The income relative deprivation affects their sleep duration by affecting their social trust,” this study constructed a mediation model through Mplus: income relative deprivation – social trust – sleep duration. And the bias correction percentile Bootstrap method is used to test the mediation effect. The results of intermediary analysis show that social trust is an important mechanism to explain the relationship between income relative deprivation and sleep duration. The income relative deprivation has a negative impact on the normal sleep duration by negatively affecting the social trust of individuals.

Third, in addition, this paper also uses three methods to test the robustness of the model: change the dependent variable, change the independent variable, and change the model, the conclusion is still stable. On the basis of instrumental variable method, the heterogeneity analysis is further carried out. The results show that the negative impact of income relative deprivation on sleep duration is only significant in eastern China, married people and groups with more than two properties. The above conclusions prove the role of income relative deprivation in maintaining normal sleep duration, and provide empirical evidence to support the adverse effects of income relative deprivation on individual normal sleep duration.

## Discussion

On the basis of combing the previous relevant literature, this study conducted an empirical analysis on the income relative deprivation and sleep duration of individuals. Previous studies on factors affecting sleep duration focused on the biomedical field, and many clinical medical research evidences fully endowed the health significance of sleep duration. However, we cannot ignore the social impact on individual behavior. The time when individuals fall asleep, wake up, and their sleep duration is determined by complex social processes. In the modern society with rapid social development, sleep problem is not only a physiological problem but also a social problem. For a long time, people believed that economic growth would bring about an increase in the income of social members and improve their living standards. However, more evidence shows that economic growth plays a stronger role in promoting the income growth of high-income groups and will exacerbate income inequality. Compared with high-income groups, low-income groups have greater psychological pressure, stronger sense of social deprivation and loss of control in life (Kim and Dimsdale, 2007; Ellaway et al., 2011). Low-income groups, out of concern about their income, will sacrifice their sleep time to obtain further education or training to increase their income potential. Or they may increase their leisure time to compensate for the loss due to low income. It can be seen that the polarization between rich and poor and income inequality brought about by economic development are not conducive to the healthy development of the whole society, and the resulting income relative deprivation will seriously affect people’s sleep duration.

### Theoretical implications

After studying the existing relevant literature, we found that there were few social studies on sleep duration. Moreover, most studies on income inequality are based on a macro-perspective and measured with Gini coefficient, which means that different social members will have the same negative feelings when facing income inequality, but this is obviously unscientific (Guoqiang et al., 2017). Lack of research is on relative deprivation of income from the micro-individual level. Sleep duration is affected by different social roles and social relationships of individuals (Meadows, 2005). Living in poverty may bring challenges to social relationships and social roles, thus reducing the possibility of individuals maintaining normal sleep duration. Therefore, different individuals have different perceptions of inequality when facing the income gap. From the perspective of relative income deprivation at the micro-individual level, this study uses empirical research to explore the impact of income inequality at the micro-level on sleep duration. To some extent, it makes up for the theoretical vacancy of the current theme.

### Practical implications

The rapid development of China’s economy has brought about serious social polarization between the rich and the poor. At the same time, the fierce social competition has also seriously affected the sleep of social members, and insufficient sleep has become a common social phenomenon. Therefore, we should give sufficient social attention to sleep duration, and it is of great practical significance to understand the factors that affect keeping normal sleep duration. According to the results of this study, we put forward the following two suggestions. First, we need to improve the income level of low-income groups and narrow the gap between the rich and the poor. The empirical results of this paper show that with the deepening of the income relative deprivation, the probability of people keeping normal sleep duration is lower. Therefore, it is very important to improve the income level of low-income groups and narrow the gap between rich and poor. Second, improve the social psychological counseling mechanism, alleviate social conflicts, and promote the establishment of a good relationship of trust between people. Our empirical study found that social trust is an important mechanism for income relative deprivation to affect individual sleep duration. The increasing income relative deprivation has a negative impact on the trust relationship between people, which is not conducive to maintaining normal sleep duration.

### Limitations and future research

This study has the following limitations, which can be further addressed in future research. First of all, due to the influence of many practical factors, this study uses cross-sectional data to study, which cannot dynamically reflect the relationship between income relative deprivation and sleep duration. In the future research, we should consider the longitudinal data analysis for many years to explore the longitudinal impact of the time change of income relative deprivation on people’s sleep duration. Second, because CFPS2018 data are adopted, the data are defined as wage income according to personal net income. Under the restriction of data conditions, we can only choose individual wage income as the index to calculate the relative deprivation of individual income, and cannot include other income except wage income. In future research, multiple databases should be merged to measure and calculate the income of social members more comprehensively. Third, because of the relative deprivation index of income in this study is measured by wage income, the sample is limited to the group with jobs, and many samples without jobs but with flexible income are eliminated, resulting in a small sample size. In future research, we should enrich the existing database, continue to expand the sample size, and make the research results more representative and generalizable.

## Data availability statement

The original contributions presented in this study are included in the article/supplementary material, further inquiries can be directed to the corresponding authors.

## Author contributions

ZP: conceptualization, methodology, software, data curation, writing – original draft preparation, and review and editing. LW: supervision, investigation, project administration, and funding acquisition. Both authors contributed to the article and approved the submitted version.
